# Probing the stoichiometry of β2-adrenergic receptor phosphorylation by targeted mass spectrometry

**DOI:** 10.1186/1750-2187-9-3

**Published:** 2014-04-01

**Authors:** Shujuan Gao, Craig Malbon, Hsien-Yu Wang

**Affiliations:** 1 Department of Pharmacology, Health Sciences Center, School of Medicine, State University of New York at Stony Brook, Stony Brook, NY 11794-8651, USA; 2 Department of Physiology & Biophysics, Health Sciences Center, School of Medicine, State University of New York at Stony Brook, Stony Brook, NY 11794-8661, USA

**Keywords:** G-protein-coupled receptors, β2-adrenergic receptor, Protein phosphorylation, Liquid chromatography–mass spectrometry/mass spectrometry (LC-MS/MS), Targeted mass spectrometry, Molar quantification, G-protein coupled receptor kinase (GRK)

## Abstract

**Background:**

Protein phosphorylation of G-protein-coupled receptors (GPCR) is central to the myriad of functions that these ubiquitous receptors perform in biology. Although readily addressable with the use of phospho-specific antibodies, analysis phosphorylation at the level of stoichiometry requires receptor isolation and advanced proteomics. We chose two key sites of potential phosphorylation of human beta2-adrenergic receptor (β2AR residues S355 and S356) to ascertain the feasibility of applying targeted mass spectrometry to establishing the stoichiometry of the phosphorylation.

**Method:**

We stimulated HEK293 cells stably expressing Flag-tagged β2AR-eGFP with 10 μM beta-adrenergic agonist (isoproterenol) and made use of proteomics and targeted mass spectrometry (MS) to quantify the molar ration of phosphorylation on S355 and S356 versus non-phosphorylated receptor in agonist-treated cells.

**Results:**

Phosphorylation of either S355 or S356 residue occurred only for agonist-occupied β2AR. The results demonstrated that pS356 is the dominant site of protein phosphorylation. The abundance of the p356 was 8.6-fold more than that of pS355. Calculation of the molar ratio of phosphorylated (pS355 plus pS356) versus non-phosphorylated receptor reveals that at high occupancy of the receptor only 12.4% of the β2AR is phosphorylated at these sites.

**Conclusions:**

Application of advanced proteomics and use of the most sensitive targeted MS strategy makes possible the detection and quantification of phosphorylation of very low abundance peptide digests of β2AR. Establishing the stoichiometry of two key sites of agonist-stimulated phosphorylation with β2AR is an essential first-step to global analysis of the stoichiometry of GPCR phosphorylation.

## Background

G-protein-coupled receptors (GPCRs) constitute a large and diverse family of intrinsic membrane proteins, all displaying heptahelical organization in the cell membrane [1]. GPCRs regulate fundamental biological processes and are the most prominent targets for drug discovery [2]. Some of the earliest successes in using GPCRs as therapeutic targets are the subfamily of adrenergic receptors [3]. In fact, adrenergic agonists and antagonists constitute the largest single class of therapeutic drugs prescribed for congestive heart failure, hypertension and asthma [1,4-6].

Protein phosphorylation of GPCRs on serinyl, threoninyl and tyrosyl residues by specific protein kinases is a prominent and essential post-translational modification (PTM) [7]. Phosphorylation plays essential roles in the down-stream signaling transduction, trafficking, and overall regulation of GPCR [8]. The β2AR has been one of two dominant model receptors (the other being the photopigment rhodopsin) employed in the detailed study of the GPCR superfamily. Upon stimulation with agonist, β2AR are phosphorylated on multiple sites by a diverse group of protein kinases, such as PKA, PKCs, and GRKs [9-14]. Phosphorylation of β2AR by GRKs recruits the important adaptor molecules β-arrestins, which in turn facilitate down-stream signaling cascades as well as clathrin-mediated endocytosis of the phosphorylated receptor [15-18].

The phosphorylation of GPCRs in general, and β2AR in particular, has been the subject of intense study for decades [19-21]. There is much left unknown about this critical PTM. Advances in generating phospho-specific antibodies enable identification of receptors phosphorylated on one or more specific residues [13]. Using such tools, regulation of the phosphorylation as well as the dephosphorylation have been probed for the β2AR and many other members of the GPCR superfamily. As empowering as this approach has been to detect GPCR phosphorylation, it cannot easily provide a key piece of information about the phosphorylation of a specific residue, namely stoichiometry. Stoichiometry is essential for the fullest understanding of the receptors, since rather large changes in phosphorylation (some reported to be 300-fold) do not reveal the mole fraction of phosphorylation. If unity (i.e., all of a specific residues are fully phosphorylated) or something substantially less (i.e., a specific residue that is essentially non-phosphorylated in the untreated state, but only 3% mole-fraction at full stimulation) much of the phosphorylation story will remain unknown. There are no easy approaches to establish these data. The best strategy is application of proteomics and advanced mass spectrometry-based techniques.

Mass spectrometry has emerged as a powerful tool to study protein PTM, especially protein phosphorylation [22,23]. More recently, emphasis has been focused on quantification of phosphorylated proteins using a variety of MS-based techniques. Quantitative phosphorproteomics using stable isotope labeling of amino acids (SILAC) in cell culture have been successfully applied to several large-scale studies [24-26]. Meanwhile, label-free strategies have been emerged that rely on the development of the advanced instrumentation and software. Notably, targeted MS is one of the most sensitive mass spectrometric strategies for quantification of peptides of interest [27,28]. Application of targeted MS to the study of rather rare membrane proteins such as the β2AR taxes the limits of the approach, but can provide mole-fraction data and insights unapproachable with indirect antibody-based analyses. In the current paper, we employed targeted MS in tandem with unlabeled dephosphorylated and phosphorylated synthetic peptides derived from sequences of the β2AR to standardize the mole-fraction phosphorylation of key β2AR residues S355 and S356. The approaches requires large-scale isolation of β2AR from cells treated with beta-adrenergic agonist.

## Results

### Phosphorylation of the β2AR

**Figure 1:**
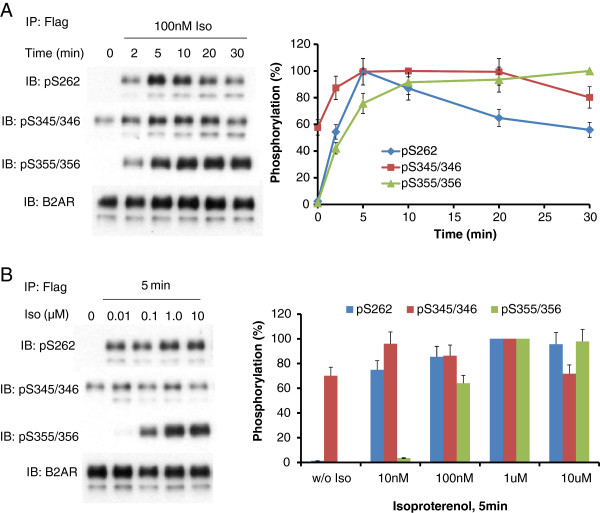
**β2AR phosphorylation in HEK293 cells challenged with beta-adrenergic agonist.** HEK293 clones stably expressing N-terminal Flag-tagged, C-terminal eGFP-tagged β2AR were treated either with 100 nM isoproterenol for the indicated times **(A)** or with varying concentrations of the beta-adrenergic agonist, isoproterenol for 5 min **(B)**. β2AR was immunoprecipitated with anti-Flag immunoadsorption beads. SDS-PAGE and immunoblotting were performed as indicated in the Materials and methods section. The phosphorylation of β2AR was normalized to total β2AR. The data were quantified and are displayed as percentage of maximum phosphorylation of each site (s). The experimental data shown is of a single analysis performed in triplicate and replicated multiple times with similar results.

β2AR are phosphorylated on distinct sites by a variety of protein kinases in cells stimulated with beta-adrenergic agonist. We initially probed for phosphorylation events on five critical residues, namely S262 (substrate for PKA), S345/S346 (substrate for PKA), and S355/S356 (substrate for GRK). HEK293 clones that were transfected and selected for stable expression of N-terminally Flag-tagged, C-terminally eGFP-tagged β2AR (Flag-β2AR-eGFP) were employed throughout. Clones were treated briefly with beta-adrenergic agonist (10 μM isoproterenol, see Figure 1). The phosphorylation of the sites displayed both a time- and a dose-dependent response to agonist treatment. In unstimulated cells, phosphorylation of S262 was very low. Upon stimulation with isoproterenol, phosphoS262 could be detected readily at 2 min. Phosphorylation of S262 was maximal at ~5 minutes, declining thereafter. Within 30 min of agonist, phosphorylation of S262 is only about 50% of maximal. S345, S346 residues showed higher basal phosphorylation compared to that of S262. Phosphorylation of S345 and S346 was observed to increase within 2 min following isoproterenol treatment, peaking at 5 min. This greater phosphorylation persisted for at least 20 min and slowly declined thereafter. The phosphorylation of GRK-specific sites, S356/S356, showed a very different pattern than those specific for PKA-catalyzed phosphorylation. Whereas basal phosphorylation was readily detected on other sites, phosphorylation of S355/S356 was not detectable in the unstimulated cells. The peak level of phosphorylation was attained within 10 min post stimulation and persisted for 30 min. Importantly, whereas phosphorylation of PKA sites was found to be maximal at 10 nM isoproterenol, phosphorylation of the GRK sites was maximal only at concentrations of isoproterenol 1.0 μM or higher (Figure 1B).

### Phosphorylation of S355/S356 is agonist occupation-dependent

**Figure 2:**
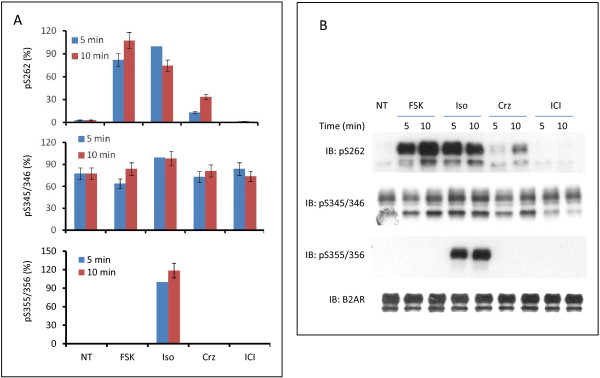
**β2AR phosphorylation in HEK293 cells challenged with beta-adrenergic agonist, forskolin, carazolol or ICI118,551.** HEK293 cells stably expressing N-terminal Flag-tagged, C-terminal eGFP-tagged β2AR treated without (NT) or with either 10 μM isoproterenol (Iso), 10 μM forskolin (FSK), 10 μM carazolol (Crz) or 10 μM ICI118,551 (ICI) for 5 or 10 min. β2AR was immunoprecipitated with anti-Flag immunoadsorption beads. SDS-PAGE and immunoblotting were performed as indicated in the Materials and methods section. The phosphorylation of β2AR was normalized to total β2AR. The data were quantified and are displayed as percentage of phosphorylation of each site (s) sampled at 5-min following stimulation with the beta-adrenergic agonist isoproterenol. The experimental data shown are of a single analysis performed in triplicate and replicated multiple times with similar results. **(A)** Quantification of phosphorylation of S262, S345/346 and S355/356. **(B)** Representative westernblotting image.

Next we probed the effects of treatment with an adenylylcyclase activator (the diterpene forskolin), a β2AR-specific antagonist (ICI118,551), and a non-selective beta-antagonist (i.e., carazolol) on the phosphorylation of β2AR S262, S345/S346, and S355/S356 (Figure 2). ICI118,551 treatment did not change the basal phosphorylation state of the receptor. Treatment with carazolol did increase the phosphorylation of S262, but not that of either S345/S346 or S355/S356. Earlier it was shown by Baker that carazolol treatment indeed stimulate intracellular accumulation of cyclic AMP in CHO cells [29]. Forskolin significantly increased the phosphorylation of S262. S262-specific phosphorylation did not decline, even after 60 min following treatment (data not shown). Interestingly, forskolin did not cause any increase of the phosphorylation of either S345/S346 or S355/S356 over basal levels. These results indicate that phosphorylation of S355/S356 was not dependent on accumulation of cyclic AMP, but rather upon the occupation of the receptor by an agonist.

### Mass spectra of phosphorylated and non-phosphorylated peptides

**Figure 3:**
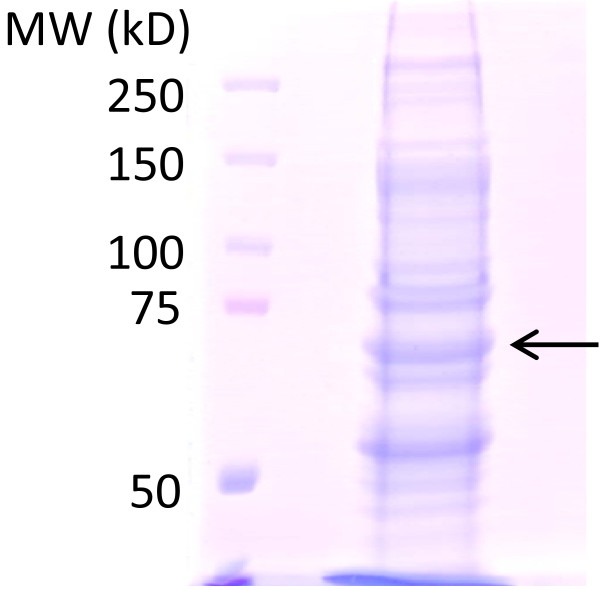
**SDS-PAGE analysis of β2AR phosphorylated in HEK293 cells challenged with beta-adrenergic agonist.** HEK293 cells stably expressing N-terminal Flag-tagged, C-terminal eGFP-tagged β2AR treated either without or with 10 μM isoproterenol for 10 min. β2AR was immunoprecipitated with anti-Flag immunoadsorption beads, treated with PNGase F, eluted from the beads, and then concentrated. Samples were subjected to SDS-PAGE on 6.5% acrylamide separating gels, as described in experimental procedures. Bands of N-terminal Flag-tagged, C-terminal eGFP-tagged β2AR are indicated with “arrows”. These data are from single experimental determinations, replicated multiple times with similar results.

**Figure 4:**
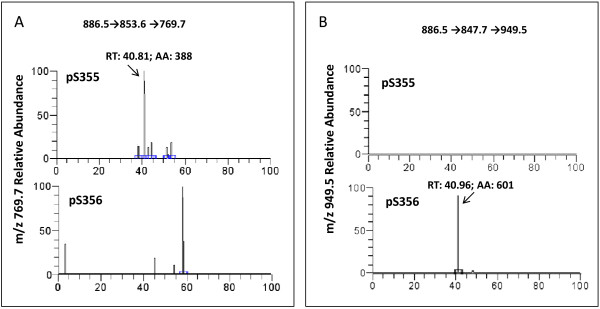
**Targeted MS of phosphorylation sites S355 and S356 of β2AR.** Relative abundance of *m/z* 769.7 **(A)** and *m/z* 949.5 **(B)** from mono-phospho-peptides, containing either pS355 or pS356. For protocol, see the Materials and methods section.

Since the phosphorylation of SS355/356 is solely dependent on the occupation of the receptor by agonist, these residues were ideally suited as targets for establishing the molar ratio of the phosphorylated versus non-phosphorylated receptor upon isoproterenol stimulation. HEK293 cells stable expressing Flag-β2AR-eGFP were stimulated with 10 μM isoproterenol in serum-free medium for 10 min and harvested. The β2AR were affinity purified from the whole-cell lysates by immunoprecipitation using anti-Flag antibody covalently crossed linked to agarose. The highly purified Flag-β2AR-eGFP then was subjected to SDS-PAGE electrophoresis (see Figure 3). The band corresponding to resolved β2AR was excised and subjected to exhaustive trypsin digestion. Phosphorylated peptides of β2AR digests detected were identified and are listed in Table 1. The mass spectra of phosphorylated and non-phosphorylated peptides containing S355/S356 residues are displayed (see Additional file 1). Peptides of each corresponding sequence to our targeted fragments were synthesized (both non- and phospho- forms) employed as standards to define both the precursor ion for MS as well as to establish ideal conditions for detecting experimental samples (see Figure 4). The precursor ion of *m/z* 859.87 is that of non-phosphorylated peptide in the MS/MS. The precursor ion of *m/z* 886.5 is that of the singly phosphorylated peptide containing either pS355 or pS356 in the MS/MS. In order to distinguish between mono-phosphopeptide containing either pS355 or pS356, two product ions from MS/MS of *m/z* 886.5 were fragmented further as indicated, 886.5→853.6 and 886.5→847.7 for MS/MS/MS. The peak at *m/z* 769.7 (886.5→853.6→769.7, y6) was the “signature” fragment for pS355 peptides (Figure4A and Additional file 2). The ion *m/z* 769.7 could not be detected with pS356 peptide at low abundance (<5 fmol). Yet the intensity of *m/z* 949.5(886.5→847.7→949.5, [y18-2H2O2+) peak representing pS356 was more than 10-fold greater than that of pS355 (Figure 4B and Additional file 3). The *m/z* 949.5(886.5→847.7→949.5, [y18-2H2O]2+) signal was robust and provided a “signature” fragment by which to quantify the abundance of the pS356 peptide of interest.

**Table 1:** Phosphorylated peptides detected by LC-MS/MS

**Peptide sequence**	**Start**	**End**
R.FHVQNL***S***QVEQDGR.T	F240	R253
R.R***S***SKFCLK.E	R260	K267
R.RS***S***KFCLK.E	R260	K267
K.AYGNGY***S***SNGNTGEQSGYHVEQEK.E	A349	K372
K.AYGNGYS***S***NGNTGEQSGYHVEQEK.E	A349	K372
K.LLCEDLPGTEDFVGHQGTVPSDNID***S***QGR.N	L376	R404

### Mass spectrometry quantification of the phosphorylation of S355/S356 residues

**Figure 5:**
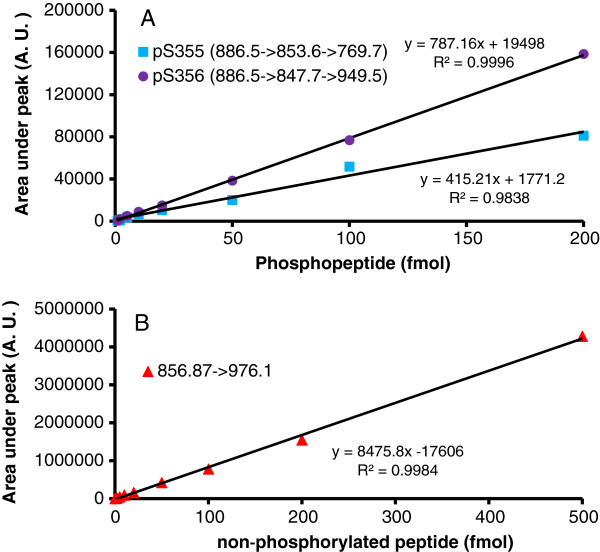
**Targeted MS of phosphorylation sites S355 and S356 of β2AR: calibration curves of target peptide standards.** Calibration curve of mono-phospho-peptides **(A)** containing pS355 (●) or pS356 (■) and non-phospho-peptide **(B)**. For protocol, see the Materials and methods section.

Ascertaining the mole-fraction of phosphorylation using MS requires creation of an internal standard of peptides of known quantities. We made use of a set of non-phosphorylated and phosphorylated synthetic peptides as standards. Analysis of these samples provided a proper calibration curve. Unknown samples then could be subjected to analysis from digests under the same conditions and then quantified using the calibration curve. For the purposes of testing the utility of this targeted MS strategy, we focused only on the signature *m/z* ion for mono-phosphopeptides containing pS355 or pS356 and non-phosphorylated peptide derived from digests of purified β2AR (Figure 5). The standard curve was linear over the range of input samples of 1 fmol to 200 fmol for the synthetic phosphopeptides. For the non-phosphorylated corresponding synthetic peptides the standard curve was linear over a range of input samples of 1 fmol to 500 fmol. Using such an approach which relies upon creating a similar standard curve for each unknown sample, we were able to quantify the area under the curve of signature ions. Representative data from isoproterenol-treated samples are shown (see Additional file 1). We calculated the mole content of each peptide (Table 2). The results can be summarized as follows for analysis of signature *m/z*: 0.56 fmol for the pS355 peptide and 4.8 fmol for the pS356 peptide were detected in targeted MS of digests from receptors isolated from cells treated with beta-adrenergic agonist. For the non-phosphorylated peptide, the targeted MS of digests of β2AR yield 43.4 fmol. These exhaustive studies permit us to make several observations about the phosphorylation of this distal region of the C-terminal tail of the β2AR. First, the S356 residue is the dominant site of β2AR phosphorylation by GRK in the cells challenged with beta-adrenergic agonist. Secondly, the S355 residue, in comparison to the S356 residue, is phosphorylated to much lesser extent in response to isoproterenol (10 μM) stimulation. The abundance of the p356 (4.8 fmol) was 8.6-fold more than that of pS355 (0.56 fmol). Finally, the mole-fraction of β2AR that is phosphorylated on these two residues, pS355 plus pS356, under these conditions (10 μM isoproterenol) is not stoichiometric. The molar ratio of phosphorylated (pS355 plus pS356) versus non-phosphorylated receptor can be calculated with confidence to be no more than 12.4% of the β2AR.

**Table 2:** Quantification of phospho- and non-phospho-peptides

**Peptides**	**fmol**	**% of total**
pS355	0.56	1.1
pS356	4.8	9.9
Non-phopsphopeptide	43.4	89.0

## Discussion

The phosphorylation (and dephosphorylation) of GPCRs are essential post-translational modifications both to signal transduction as well as to trafficking and to regulation of GPCRs involved in a signal propagation event. In the present study, we have used LC-MS/MS/MS in an effort to quantify the phosphorylation of β2AR following agonist stimulation at one key set of residues. PKA, PKC, and GRK all catalyze the phosphorylation of members of the superfamily of GPCRs, including the well-studied β2AR. It is known that the region of the C-terminal tail of the β2AR that encompasses residues S355/S356 is phosphorylated by GRK in cells challenged with beta-adrenergic agonist. The central role of GRK in GPCR biology [30-32] is not the only reason we employed this region for testing the targeted MS strategy. The phosphorylation of the peptides that harbor S355/S356 is rather unique. In the absence of agonist treatment virtually no phosphorylation is detected (this report and other study [10]). This basal, non-phosphorylated state of this region of the β2AR was revealed by immunoblotting with antibodies specific for phosphorylation of either of the residues. Our own MS analysis confirmed the results of the immunoblotting, *i.e.,* the S355 and S356 residues clearly are non-phosphorylated in the absence of agonist. This unique feature of S355/S356 sites being phosphorylated only when the receptor is occupied by agonist has important implications for understanding phosphorylation of the β2AR by GRK, versus that by PKA or by PKC. We demonstrated the final criterion for selection in the time-courses of β2AR phosphorylation. Not only is the phosphorylation of the S355/S356 residues robust in response to stimulation of cells with isoproterenol, but this phosphorylation, unlike that of S262 by PKA, is relatively stable over a longer time-frame. Thus our focus on these sites of potential phosphorylation rather than others was intentional and well placed. S355/S356 were best suited for testing the ability of targeted MS to analyze fragments from low abundance proteins that display multiple sites of potential phosphorylation by more than one protein kinase.

Another method employed by others to detect protein phosphorylation by MS is based upon prior enrichment of phosphopeptides using affinity chromatography on matrices with immobilized metal ions. Using this method, phosphopeptides of the β2AR have been isolated and studied [33,34]. This approach suffers methodologically, however, by virtue of its inability to quantify the non-phosphorylated peptide counterparts in a receptor digest. Likewise, it cannot accurately assess yield of the peptides. Rather this type of strategy either ignores or is forced to calculate only “relative” change. To accomplish this, the strategy assumes that the yields must be the same for all peptides, which is not a valid assumption. Thus, as with immunoblotting approach, affinity chromatography of phosphopeptides cannot yield insights on stoichiometry of phosphorylation. This is a major limitation, since without the denominator of the fraction, an observed three-fold relative change may represent either a change from a baseline of 10% to 30% or from a baseline of 1% to 3%. The inability to establish the stoichiometry of interacting partners in solid-state signaling paradigms like G-protein-coupled signaling constitutes a major stumbling block to enabling a fuller understanding of the overall signaling pathways.

Our goal was to test if we could detect the mole-fraction of phosphorylation events in GPCRs, using the β2AR as the test case. More specifically we sought to identify and quantify the phosphopeptides vs non-phosphorylated peptides (the sum then constituting the total, or denominator) within one experimental sample. We made good use of targeted MS, which allows the quantification of low-abundance analytes of interest from a complex mixture empowered by the MS [27,28]. We made good use of synthetic peptides with the same sequence (both non-phosphorylated and phosphor- forms) corresponding to the digest products of the target peptides of interest. We were able to probe and to characterize the analytical methods to be employed with unknown samples isolated from cells treated with (stimulated) beta-adrenergic agonist first using standards. Standard curves of non-phosphopeptides and phosphopeptides derived from β2AR were created, calibrated, and then employed with unknown samples from digests of β2AR isolated from isoproterenol-treated cells. In full spectra scan, the potentially doubly-phosphorylated peptide of pS355pS356 was not in fact observed. This might be due to the relative abundance of the doubly-phosphorylated vicinal serine residues in the peptide being too low for detection by this high-resolution instrument. Statherin reported the sequence for a salivary phosphoprotein containing two vicinal phosphoserine residues (Ser2-Ser3) each phosphorylated [35]. Vicinal tyrosine residues have been reported in a *Haemophilus* influenza ferric-binding protein, but their function as substrates for a tyrosine kinase is not known [36]. It would not at all be unlikely that phosphorylation of one of the pairs of vicinal serines, theonines, or tyrosines virtually precludes sterically a second phosphorylation event. If true, the S356 appears to the preferred substrate, which once phosphorylated, precludes phosphorylation of the other vicinal serine residue. Even if the vicinal doubly-phosphorylated species (i.e., pS355, pS356) exists, its abundance must be very much lower than either singly-phosphorylated species.

A comment about the absolute mole-fraction of phosphorylation of the peptide harboring the S355/S356 residues seems in order. Should we expect a mole-fraction equivalent of phosphorylation of either peptide? High mole-fraction phosphorylation would seem to defeat one important feature of signaling, i.e., dynamic range of signaling. Many phosphorylation events operate in the same range as that reported here. What is unique about the GRK-catalyzed phosphorylation of these sites is the apparently absolute dependence of the phosphorylation upon receptor occupancy. Since we extract the entire β2AR complement from the cells, the mole-fraction must include some significant fraction of receptor that is not poised to interact with agonist (e.g., internalized). Assuming half of β2AR are not accessible to the bulk solution of the cell culture media would elevate the mole-fraction to only about one-quarter of the cell-surface complement. Yet the amplification or gain of G-protein-based signaling is sufficient that the full downstream response to beta-adrenergic agonist may only require this submaximal level of phosphorylation. Differential losses between non-phosphorylated versus phosphorylated β2AR derived fragments of interest might also affect the amount of peptides detected. For example, in-gel digestion of the β2AR which is the protocol, might display differential release of non-phosphorylated versus phosphorylated peptides from the gel. Although possible, our experience to date does not support it. We cannot rule out that some other post-translational modifications that are differentially effected by the presence of one or more phosphate groups, such as ubiquitination [37] at either K348 or K372 might alter protease digestion patterns of the β2AR. Such post-translational modifications might generate longer aberrant peptides, which might be missed or not fully appreciated in the analysis. Speculation aside, our results are the first to demonstrate feasibility of targeted MS strategy to establish the mole-fraction of the phosphorylation of a low abundance GPCR. In this case the substrate was the β2AR and GRK was the kinase of interest. Successful application of the targeted MS technique to study low abundance, intrinsic membrane proteins of the nature of the seven transmembrane segmented GPCR offers substantiated promise. Targeted MS may well be the only strategy that can reveal the fine details of the stoichiometry of protein phosphorylation that are essential to our understanding of signal transduction.

## Materials and methods

### Materials

The following peptides corresponding to A349-K372 of the human β2AR were synthesized by United BioSystems (Herdon, VA): A349-K372 (AYGNGYSSNGNTGE QSGYHVEQEK), A349-K372pS355 (AYGNGY (pS) SNGNTGEQSGYHVEQEK), and A349-K372pS356 (AYGNGYS (pS) NGNTGEQSGYHVEQEK). Mouse monoclonal antibodies against phospho-S262 peptides were a gift from Dr. Richard B. Clark (The University of Texas, Houston). Rabbit anti-β2AR, rabbit anti-phospho-serine 345/346, and rabbit anti-phospho-serine 355/356 antibodies were purchased from Santa Cruz Biotechnology (Dallas, TX). Rabbit anti-mouse IgG-peroxidase labeled was purchased from Sigma (St. Louis, MO). Goat anti-rabbit IgG-peroxidase labeled was purchased from KPL (Gaithersburg, MD).

### Cell culture and treatment with beta-adrenergic agonist

HEK293 clones stably expressing human β2AR with N-terminal Flag-tag, C-terminal eGFP-tag (Flag-hβ2AR-eGFP) were created via transfection of HEK293 cell with plasmid harboring the coding sequence for Flag-hβ2AR-eGFP. Cells with membrane eGFP were selected (under fluorescence microscope) and then subcultured for these experiments. HEK293 clones expressing the tagged β2AR were grown in Dulbecco’s Modified Eagle Medium (DMEM) containing 10% (v/v) fetal bovine serum, 100 μg/ml penicillin, 100 μg/ml streptomycin and 2 mM L-glutamine, and maintained in 5% CO_2_/95% air at 37°C. Cells were grown to 90% confluence in 100 mm-dishes, then washed and replenished with DMEM pre-warmed to 37°C. Beta-adrenergic agonists or antagonists were added cell culture media to a final concentration of 10 μM (unless otherwise specified). The cells were incubated for indicated times. Control dishes were incubated with DMEM only, without agonists or antagonists. Cells were harvested with 120 μl cell lysis buffer (phosphate-buffered saline containing 10 mM NaF, 10 mM sodium pyrophosphate, 0.2 mM sodium vanadate, 1% n-dodecyl-β-D-maltoside, 2 × protease inhibitor tablet (EDTA-free, Roche, Nutley, NJ), 2 × phos-STOP phosphatase inhibitor cocktail tablet (Roche, Nutley, NJ). Whole cell lysates were stored at −80°C prior to analysis.

### Immunoprecipitation of β2AR and SDS-PAGE

Cell lysates were centrifuged at 14,000 rpm for 15 min, and the supernatants were collected. Routinely ~50 mg protein of supernatant samples (13 mg/ml) was incubated with 200 μl of anti-Flagbeads (Sigma, St. Louis, MO) for 6 h at 4°C with constant rotation. At the end of the incubation, the affinity matrix beads were washed with phosphate-buffered saline (PBS) for three times and then incubated with 5.0 μl of PNGase F (New England Biolabs, Ipswich MA) in reaction buffer at 37°C for 1 h. The mixture was incubated overnight in the presence of elution buffer containing 60 mM Tris, pH 6.8, 2% SDS, 2% 2-mecaptoethanol, 10% glycerol, and 0.01% bromophenol blue to elute proteins adsorbed to the affinity matrix. The beads were collected by centrifugation at 3,000 rpm for 1 min. The supernatant was collected and applied to a Microcon YM30 centrifuge filter unit and subjected to centrifugation at 5,000 × *g* for 25 min. The concentrated sample then was subjected to SDS-polyacrylamide gel electrophoresis (SDS-PAGE) on 6.5% polyacrylamide gels, resolved at 110 V over 90 min. Proteins in gel were stained with Coomassie brilliant blue and then destained with 30% ethanol and 10% acetic acid. Protein bands corresponding to the known molecular weight of β2AR were excised and then subjected to exhaustive digestion with trypsin.

### Immunoblotting

For immuboblotting, protein samples were immunoprecipitated as described. The bound protein was eluted directly from the affinity matrix beads without PNGase F treatment. The eluted proteins were resolved on 7% SDS-PAGE and transferred electrophoretically to polyvinylidene difluoride membranes. Membranes were probed with one of the following anidodies, anti-β2AR (1: 6,000 dilution), anti-pS262 (1:2,000 dilution), anti-pS345/346 (1:1,000 dilution) and anti-pS355/356 (1:3,000 dilution) antibodies prepared in PBST (PBS with 0.05% Tween 20) at room temperature for 1 h. The membranes were washed with PBST for 10 min and the process was repeated for three times in close succession. Horseradish peroxidase-labeled secondary antibodies then were added and the mixture incubated for 45 min. The membranes were washed with PBST for 20 min, three times in succession. The resolved bands were visualized with enhanced chemiluminescence plus. The images were quantified with Image*quant* software (GE Healthcare Life Sciences, Piscataway, NJ). The data were normalized against the signal of β2AR obtained with anti-β2AR antibody.

### Sample preparation, proteomics, and MS-based analyses

The gel lane containing β2AR was excised and the protein reduced with DTT, alkylated with iodoacetamide, and then digested with MS-grade trypsin (Mass Spectrometry Grade Promega Gold; Promega, Fitchburg, WI), following the protocol of Shevchenko *et al.*[38] with minor modifications. The resulting peptide digest was dissolved with 2% acetonitrile containing 0.1% formic acid (solution A) for exhaustive analysis. Half of the peptide mixture was analyzed by automated microcaplillary liquid chromatography-tandem mass spectrometry. The fused-silica microcapillaries column (100 μm inner diameter - i.d.by 10 cm) was packed in-house with Magic C8 material (5 μm particles, Michrom, Auburn, CA). The column was installed in-line with Dionex 3000 HPLC pump running at 500 nL min^−1^. The peptides were loaded with an autosampler and eluted from the column by applying a 35 min gradient of 2% solution B (98% acetonitrile, 0.1% formic acid) to 40% solution B. The gradient was switched from 40% to 80% solution B over 3 min and held constant for 8 min. Finally, the gradient was changed from 80% buffer B to 100% solution A over 0.1 min, and then held constant at 100% solution A for 19 more minutes. The application of a 2.0 kV distal voltage then electrosprayed the eluting peptides directly into an LTQ XL ion trap mass spectrometer equipped with a nano-liquid chromatography electrospray ionization source. Full mass spectra (MS) were recorded on the peptides over a 400 to 2000 *m/z* range followed by MS/MS of *m/z* 859.87 for the non-phosphorylated peptide, MS/MS of *m/z* 886.5 for the singly phosphorylated peptide, MS/MS/MS of *m/z* 886.5 → 853.6 for the fragment of the phosphorylated peptide that lost phosphoric acid, and MS/MS/MS of *m/z* 886.5 → 847.7 for the fragment of the phosphorylated peptide that lost water in addition to the phosphoric acid. These respective fragments of the MS/MS and MS/MS/MS scans were plotted in Xcalibur software manually and the area under curve recorded and quantified. A calibration curve was constructed with samples containing 1 fmol, 2 fmol, 5 fmol, 10 fmol, 20 fmol, 50 fmol and 100 fmol (or in some cases 500 fmol) of the respective peptides added to 100 ng protein of an *E.coli* trypsin digest.

The results of the experiments (extending from whole-cell extractions to full targeted MS analysis) were highly reproducible and were replicated multiple times.

## Abbreviations

PKA: Protein kinase A; PKC: Protein kinase C; GRK: G-protein-coupled receptor kinase; GPCR: G-protein-coupled receptor; β2AR: Beta2-adrenergic receptor; HEK: Human embryonic kidney; eGFP: Enhanced green fluorescent protein; Flag: Octapeptide sequence DYKDDDDK; Flag-β2AR-eGFP: N-terminal Flag-tagged, C-terminal eGFP-tagged human β2AR; PBS: Phosphate-buffed saline; SDS-PAGE: Sodium dodecyl sulfate-polyacrylamide gel electrophoresis; SILAC: Stable isotope labeling of amino acids.

## Competing interests

The authors declare that they have no competing interests.

## Authors’ contributions

SG, CCM, and HYW jointly designed the study. SG executed the experiments and analyzed the proteomics with HYW and CCM. SG first authored the manuscript, while CCM and HYW provided support, assistance with and editing of the manuscript. All authors read and approved the final manuscript.

## Supplementary Material

Additional file 1 **Phospho-peptides and non-phosphorylated peptides detected in β2AR isolated from cells treated with beta-adrenergic agonist.** Samples of digests of purified β2AR were subjected to either LC-MS/MS/MS or LC-MS/MS. (A), display of spectra of signature ion *m/z* 769.7 detected in the sample and the area under curve at RT = 40.36 min for pS355 peptide (A349-K372). (B), display of spectra of signature ion *m/z* 949.7 detected in the sample and the area under curve at RT = 40.36 min for pS356 peptide (A349-K372). (C), display of the spectra of signature ion *m/z* 976.5 detected in the sample and the area under curve at RT = 40.35 min for non-phosphorylated peptide (A349-K372). The data shown are of a single analysis, replicated multiple times with identical results. For protocol, see the Materials and methods section. Click here for file

Additional file 2 **MS/MS/MS spectra of fragmented peptide of *m/z *886.5 → 853.6.** (A), representative MS/MS/MS fragmentation spectra of *m/z* 886.5 → 853.6. for peptide containing phosphorylated S355; (B) representative MS/MS/MS fragmentation spectra of *m/z* 886.5 → 853.6. for peptide containing phosphorylated S356. The phosphorylated sites are highlighted in red. The data shown are of a single analysis, replicated multiple times with identical results. For protocol, see the Materials and methods section. Click here for file

Additional file 3 **MS/MS/MS spectra of fragmented peptide of *m/z *886.5 → 847.7.** (A), representative MS/MS/MS fragmentation spectra of *m/z* 886.5 → 847.7. for peptide containing phosphorylated S356. (B), representative MS/MS/MS fragmentation spectra of *m/z* 886.5 → 847.7 for peptide containing phosphorylated S356. The phosphorylated sites are highlighted in red. The data shown are of a single analysis, replicated multiple times with identical results. For protocol, see the Materials and methods section. Click here for file
