# Supplementary material for: Probing the stoichiometry of β2-adrenergic receptor phosphorylation by targeted mass spectrometry
Source: J Mol Signal. 2014 Apr 1;9:3. doi: 10.1186/1750-2187-9-3 (PMC4022239; doi:10.1186/1750-2187-9-3)

A

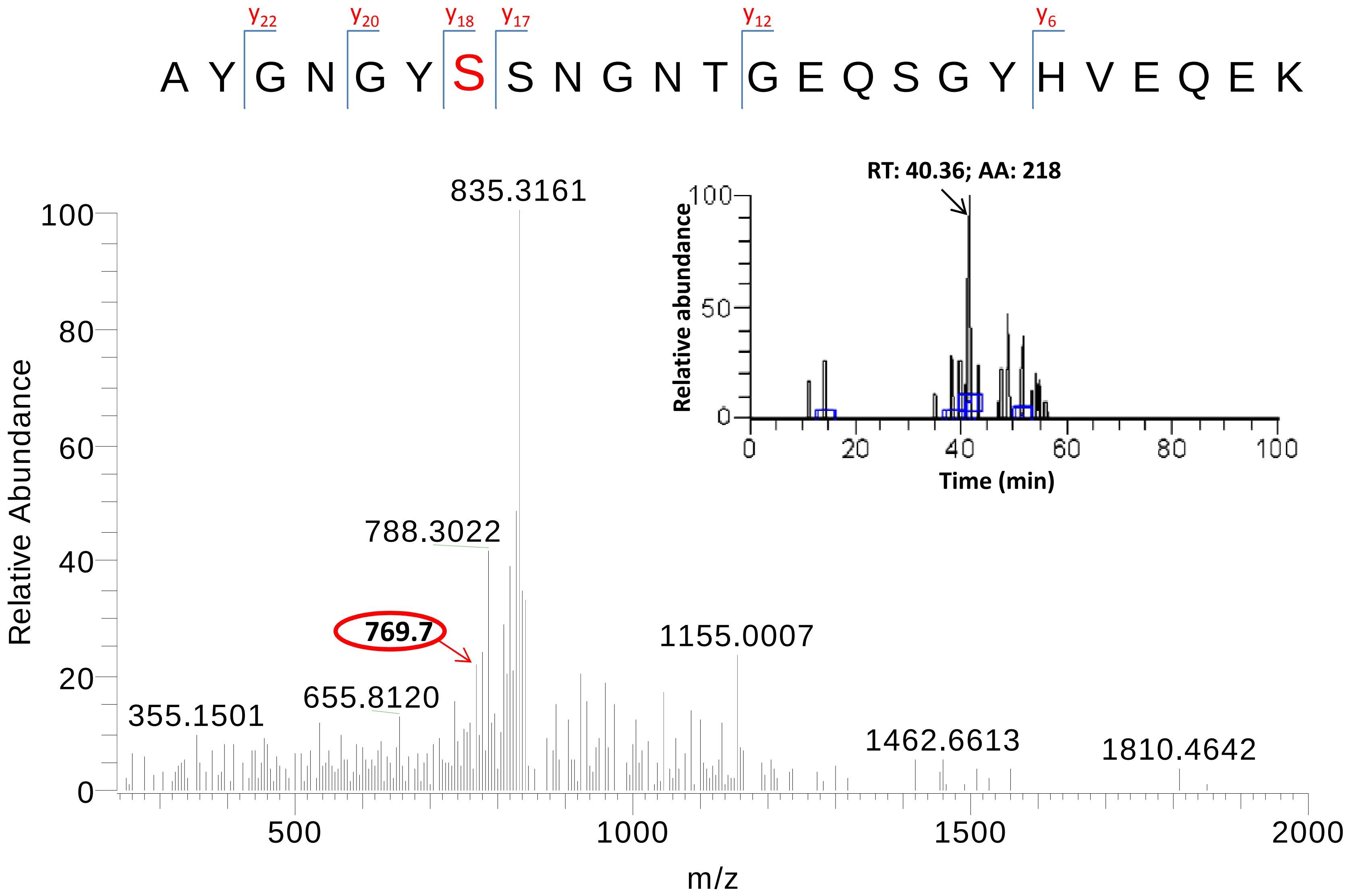
y22
y20
y18
y17
y12
y6
A Y G N G Y S S N G N T G E Q S G Y H V E Q E K
835.3161
788.3022
769.7
655.8120
355.1501
1155.0007
1462.6613
1810.4642
Relative Abundance
m/z
RT: 40.36; AA: 218
Relative abundance
Time (min)

Additional file 3

B

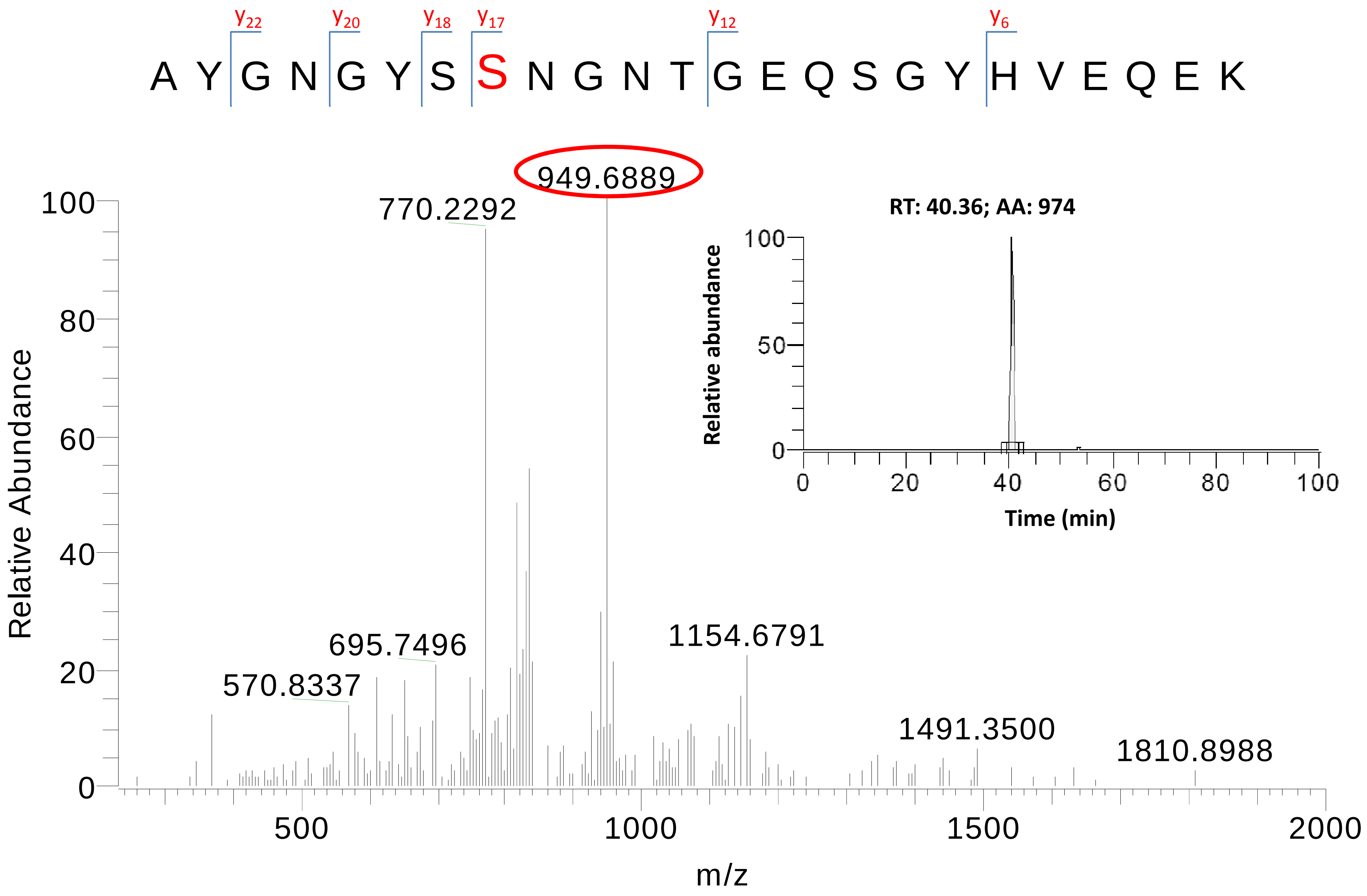

C

A Y G N G Y S S N G N T G E Q S G Y H V E Q E K

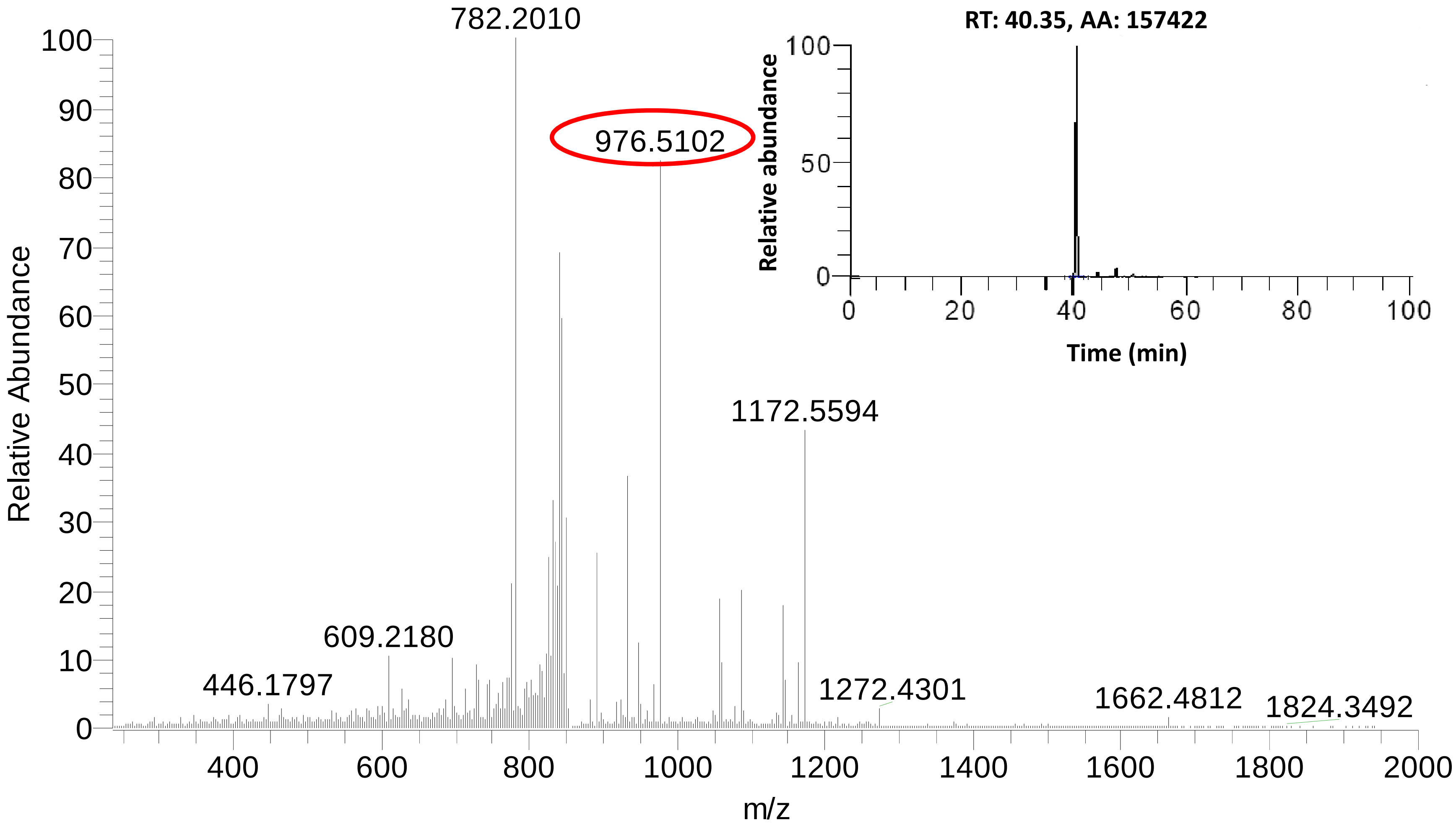

Supplement: Additional file 1 — Phospho-peptides and non-phosphorylated peptides detected in β2AR isolated from cells treated with beta-adrenergic agonist. Samples of digests of purified β2AR were subjected to either LC-MS/MS/MS or LC-MS/MS. (A), display of spectra of signature ion m/z 769.7 detected in the sample and the area under curve at RT = 40.36 min for pS355 peptide (A349-K372). (B), display of spectra of signature ion m/z 949.7 detected in the sample and the area under curve at RT = 40.36 min for pS356 peptide (A349-K372). (C), display of the spectra of signature ion m/z 976.5 detected in the sample and the area under curve at RT = 40.35 min for non-phosphorylated peptide (A349-K372). The data shown are of a single analysis, replicated multiple times with identical results. For protocol, see the Materials and methods section. [file 1750-2187-9-3-S1.pdf]
