# Supplementary figures and images for: Probing the stoichiometry of β2-adrenergic receptor phosphorylation by targeted mass spectrometry
Source: J Mol Signal. 2014 Apr 1;9:3. doi: 10.1186/1750-2187-9-3 (PMC4022239; doi:10.1186/1750-2187-9-3)

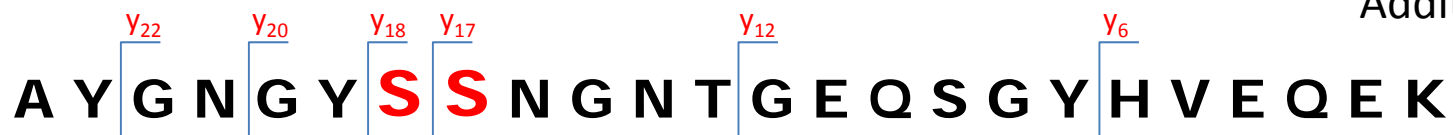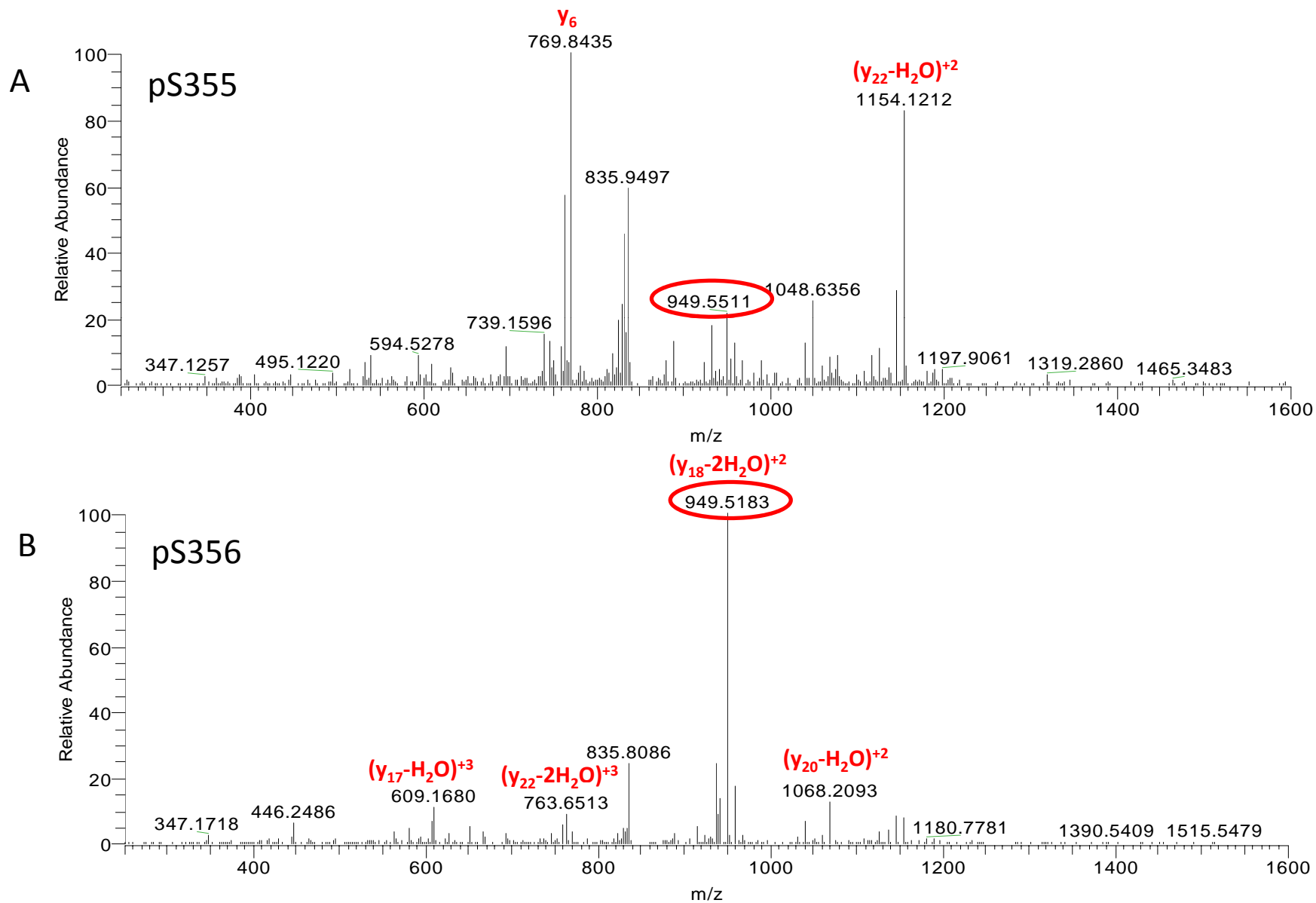

Supplement: Additional file 3 — MS/MS/MS spectra of fragmented peptide of m/z 886.5 → 847.7. (A), representative MS/MS/MS fragmentation spectra of m/z 886.5 → 847.7. for peptide containing phosphorylated S356. (B), representative MS/MS/MS fragmentation spectra of m/z 886.5 → 847.7 for peptide containing phosphorylated S356. The phosphorylated sites are highlighted in red. The data shown are of a single analysis, replicated multiple times with identical results. For protocol, see the Materials and methods section. [file 1750-2187-9-3-S3.pdf]
